# Binge-watching and gender-specific effects on academic, social, and mental well-being in children and adolescents

**DOI:** 10.1371/journal.pone.0329655

**Published:** 2025-08-22

**Authors:** Nick Tse, Natalie Sze-Nga Pang, Xin Wang, Yiran Li, Camilla Kin-Ming Lo, Xue Yang

**Affiliations:** 1 Department of Applied Social Sciences, HKCT Institute of Higher Education, Hong Kong SAR, China; 2 The Jockey Club School of Public Health and Primary Care, Faculty of Medicine, Chinese University of Hong Kong, Hong Kong SAR, China; 3 Department of Applied Social Sciences, The Hong Kong Polytechnic University, Hong Kong SAR, China; Health Researcher, SPAIN

## Abstract

**Introduction:**

Binge-watching refers to the consumption of screen-based content (excluding gaming) for five or more consecutive hours in a single session within the past month, which is an emerging behavior of concern among students. This study aims to examine the rate of binge-watching among children and adolescents in Hong Kong as well as the potential association with their psychological, social, and educational self-efficacy.

**Methods:**

A cross-sectional survey was conducted with 2,267 students from primary and secondary schools in Hong Kong, recruited through convenience sampling. The survey assessed levels of binge-watching, mental health, sleep quality, social support, loneliness, and educational self-efficacy. Linear regression analyses were performed, both with and without adjustments for age and watching time by gender.

**Results:**

The findings revealed that 47.1% of the participants (45.1% males and 49.3% females) engaged in binge-watching at least once in the past month. Binge-watching was positively associated with depression, anxiety, stress, and loneliness, but negatively associated with educational self-efficacy in both genders (*p* < .05). Gender-specific effects were found that binge-watching was significantly associated with poorer sleep quality in males (*p* < .001), but not in females (*p* > .05). In contrast, a significant negative association between binge-watching and social support was found in females (*p* < .01), but not in males.

**Conclusion:**

This study emphasizes the significant rate of problematic watching patterns and various impacts. Early identification and early prevention are warranted.

## Introduction

Binge-watching has become prevalent due to the rise of over-the-top (OTT) platforms, such as Netflix, Disney + , Amazon Prime Video, HBO Max, Apple TV, Baidu, and Tencent Video [1] as well as reels and short videos on social media platforms like TikTok, Instagram, and YouTube. Moreover, binge-watching has increased dramatically and has been normalized during the COVID-19 quarantine. A recent study reported that approximately 70% of adolescents aged 15–19 years binge-watched TV series during the COVID-19 quarantine period [2]. Binge-watching is prevalent in all age groups, particularly among the youth [3,4]. However, studies on binge-watching in children and adolescents are still lacking. Kumar, Goyal [5] conducted an online survey among 123 secondary students in Chandigarh (aged 14–18 years) during the COVID-19 pandemic and found that 13% watched more than eight episodes of TV shows and over four hours in one sitting.

The definition of binge-watching continues to change as researchers develop different definitions based on various criteria. The term ‘binge’ refers to excessive consumption and time spent on an activity, such as binge eating or binge drinking [6]. Multiple definitions have been proposed to operationalize binge-watching as the consecutive viewing of multiple episodes of television episodes in one sitting or the measurement of viewing duration and pattern [4,7–12]. The definition of binge-watching according to Netflix and several scholars have suggested as viewing two or more consecutive episodes of a specific series in one sitting and consuming the entire season in a single day. Recent studies also define binge-watching through three core essential components which include watching time duration, episode quantity, and continuous viewing [1,7,12–16].

The current study defined binge-watching as continuously engaging in five or more hours of screen viewing (excluding gaming) in the past month, based on the concept of binge-gaming in the most recent study [17]. This definition effectively captures key characteristics of binge-watching through duration, continuity, and repetition, while preventing ambiguous measurements of each TV episode or short video lengths. The definition provides consistent and reliable measurement which enables future research to achieve better operationalization.

### Binge-watching and psychological impact

Binge-watching offers instant gratification through escape but leads to long-term psychological problems which include anxiety, depression, and sleep disturbances. However, most studies have been conducted in adult populations [18–20], with a recent systematic review and meta-analysis found moderate correlations between binge-watching and stress, anxiety and depression [21,22]. The study by Özkent and Açıkel [3] was the only survey conducted among adolescents, which revealed that 60.3% of 189 Turkish adolescents were identified as binge-watchers, and this group demonstrated higher levels of emotional problems.

### Social impact

The motives behind binge-watching often include the need for social interaction and the fear of missing out (FOMO) [23–25]. Binge-watchers demonstrate higher levels of prosocial engagement with fictional characters and are more likely to be opinion leaders in their peers [15,25]. A positive correlation between binge-watching and loneliness was found in an adolescent study in India [26]. Binge-watching and prolonged screen time may reduce the time spent on face-to-face communication with families and friends, potentially leading to parent-child conflicts [27,28]. Research has also demonstrated a consistently negative relationship between prolonged screen time and received/perceived social support [29,30].

### Academic impact

Binge-watching can be particularly harmful to student’s academic performance and educational self-efficacy, also known as academic self-efficacy, which refers to students’ perception of their ability to plan and execute actions to complete academic tasks and achieve their goals [31]. Excessive binge-watching may damage self-regulation while reducing focus and, lowering self-confidence in academic performances, and increasing the risk of academic procrastination [32–35]. Research conducted on Indian students between 16 and 25 years old found that heavy use of OTT platforms negatively affected their academic performance, concentration, productivity, health, and time management [36]. The growing trend of binge-watching among children and adolescents and its specific impacts on psychological, social, and academic outcomes remain limited understood.

### Gender differences

Gender differences in the role of binge-watching remain inconclusive in previous literature. It has been suggested that binge-watching is gender-neutral, with the major difference being the preference for content [37]. Males tend to prefer science fiction, while females are more inclined towards comedies and dramas [4], emerging research indicates potential gender-specific motivations and impacts. A recent study of 300 Pakistani teenagers aged 13–19 years showed no significant gender differences in binge-watching duration, and both genders watched for entertainment, escape, and excitement. However, the study revealed different motivations and psychological impacts between genders. Female binge-watchers were predominantly driven by FOMO and social interaction needs, yet males were motivated by excitement. Female binge-watchers were also more likely to experience sleep disturbances, while male binge-watchers who showed decreased productivity [38]. Thus, it is postulated that the relationship between binge-watching and psychological, social, and educational self-efficacy will be influenced by gender differences. However, these gender-specific influences, particularly among children and adolescents, remain largely unexplored.

### The present study

Most of the existing studies have concentrated on college students and adults. However, studies on binge-watching in children and adolescents are lacking. To fill this research gap, this study tested the roles of binge-watching in various aspects of functioning (mental, social, and academic) and its potential gender differences in a large-scale sample of children and adolescents in Hong Kong. Our hypotheses included:

**H1:** Binge-watching would be positively associated with mental health problems (i.e., depression, anxiety, stress, and sleep disturbances).

**H2:** Binge-watching would be negatively associated with educational self-efficacy.

**H3:** Binge-watching would be negatively associated with perceived social support and positively associated with loneliness.

**H4:** The strength of these associations may vary by gender

## Materials and methods

### Study design and participants

A cross-sectional school-based survey was conducted from June to December 2022, excluding July and August, which were summer school holidays in six primary schools (*n* = 1,491) and four secondary schools (*n* = 776) in Hong Kong, with a convenience sampling. In total, 2,267 valid respondents participated in this study. The inclusion criteria included (a) being primary or secondary school students, (b) being willing to participate, (c) providing informed consent from both students and their parents, and (d) being Chinese speakers.

### Recruitment procedures

The research team collaborated with a professional alliance for school counseling to distribute invitation letters, introducing the study to all government-funded primary (*n* = 422) and secondary (*n* = 390) schools in Hong Kong. Schools interested in participating were instructed to contact the research team for details on obtaining parental consent. Trained research personnel, independent from the research team, facilitated the survey in the classroom, following standardized protocols provided by the research team. The survey took 15–20 minutes to complete. Participants were guaranteed the principles of voluntariness, anonymity, and confidentiality. No incentives were provided to participants.

### Ethical considerations

Informed consent was obtained from both the student and their parents. This study did not involve any clinical trials; no patient data was collected, and no identifiable student data was obtained. All procedures took place according to the Declaration of Helsinki. Ethical approval was granted by the Survey and Behavioral Ethics Committee of the corresponding author’s university (SBRE-21–0731).

### Study instruments

#### Binge-watching.

Binge-watching was defined as engaging in five or more consecutive hours of screen-watching on social media without interruptions in one session. Participants were asked if they had engaged in binge-watching behavior in the past month (0 = *No*, 1 = *Yes*). This survey adopted an inclusive definition of binge-watching and assessed it by quantifying the hours of screen time, which refers to the criteria established for identifying binge-gaming [17].

#### Academic outcome.

The **Educational Self-Efficacy Scale (ESES)** was used to assess participants’ perceived ability to learn and attain academic outcomes. The scale consists of five items measured on a five-point Likert scale, with 1 = *Not at all confident* and 5 = *Extremely confident*. Higher scores indicate having more confidence in achieving academic outcomes [39]. Cronbach’s alpha was.89.

#### Social outcomes.

The **Multidimensional Scale of Perceived Social Support (MSPSS)** was used to measure participants’ perceived social support from family, friends, and significant others [40]. It consists of 12 items measured on a 7-point Likert scale: 1 = *Very strongly disagree* to 7 = *Strongly agree*. The higher score indicates a higher level of perceived social support. The Chinese version of MSPSS demonstrated a high internal consistency, with a Cronbach’s alpha coefficient of.89 [41]. Cronbach’s alpha for this study was estimated to be.93.

The **UCLA Loneliness Scale** was used to measure the feelings of loneliness or social isolation. Items were measured on a 3-point Likert scale of 1 = *Hardly ever*, 2 = *Some of the time*, and 3 = *Often*. The scale has good reliability and validity (Cronbach’s alpha is.72–.87) [42]. Cronbach’s alpha was.82 in the current study.

#### Mental outcomes.

The **Depression Anxiety and Stress Scale (DASS-21)** was used to assess participants’ levels of depression, anxiety, and stress over the past week [43]. Items were measured on a Likert scale ranging from 0 = *Did not apply to me at all* to 3 = *Applied to me very much or most of the time*. The cutoffs were determined by multiplying the total score in each subscale by two; the cutoffs for depression, anxiety, and stress are 10, 8, and 15, respectively [44]. This scale is well-validated among Chinese populations and has good reliability and validity [44]. Cronbach’s alpha in this study was.93.

The **Pittsburgh Sleep Quality Index (PSQI)** was used to measure participants’ sleep quality in the past four weeks. This scale is rated on a Likert scale (0 = *No difficulty* to 3 = *Severe difficulty*). A higher score indicates a lower quality of sleep. Individuals who scored five or greater are considered to have a sleep disorder. The PSQI demonstrates good reliability and validity [45]. Cronbach’s alpha for this study was.73.

#### Background variables.

The information of gender, age, and average watching hours per day during weekdays and weekends was reported by the participants.

### Statistical analysis

The participants’ demographic information, including gender, age, time spent watching, mental outcomes, social outcomes, academic outcomes, and the percentage of participants engaging in binge-watching, was described. Univariate and adjusted (adjusted for age and average watching time) linear regression models were conducted to test the associations between binge-watching and well-being outcomes, including depression, anxiety, stress, sleep quality, loneliness, social support, and educational self-efficacy. SPSS version 28 was used for data analyses. Statistical significance was set at a *p*-value below.05.

Two regression models were tested. Model 1 reported unadjusted standardized coefficients to explore the raw relationships between binge-watching and psychosocial outcomes. Model 2 included age and average daily screen-viewing time as covariates to adjust for potential confounding factors and to provide a more controlled understanding of these relationships.

## Results

The total sample size for analyses was 2,267, including 1,228 males (*M* = 12.05, *SD* = 2.35) and 1,039 females (*M* = 11.81, *SD* = 2.39) in the S1 Table. In total, 554 boys (45.1%) and 512 girls (49.3%) were classified as binge-watchers in this study. Females reported more binge watching, anxiety, and poorer sleep quality than males.

In univariate linear regression analyses, binge-watching was significantly associated with all mental, social, and educational outcomes in both genders (*p* < .05), except for social support in males. Adjusted linear regression analyses revealed that binge-watching was positively associated with depression, anxiety, stress, and loneliness while being negatively associated with educational self-efficacy in both genders. Binge-watching was significantly associated with poor sleep quality in males but not in females. For females, binge-watching was significantly negatively associated with social support, but not for males in the S2 Table.

## Discussion

This study discovered that binge-watching was associated with mental distress (depressive, anxiety, and stress symptoms), lower educational self-efficacy, and reduced social support among children and adolescents. The survey reported nearly half of the students engaged in binge-watching in the past month, reflecting that binge-watching is quite common among this population. These findings generally support our hypotheses and reveal interesting gender-specific patterns. In particular, binge-watching was linked to poor sleep quality in males and lower levels of perceived social support in females, which indicates different aspects of vulnerability. The study stands as one of the initial extensive investigations which explore binge-watching relationships with various aspects of child and adolescent well-being.

This study revealed a negative correlation in both male and female populations. These findings match previous studies which demonstrated that binge-watching negatively affected academic performance and productivity [34–36] and extend its potential effect on students’ beliefs and self-confidence in their ability to perform academic tasks. Although evidence among children and adolescents remains limited, several studies with college students provide insight into potential mechanisms underlying this relationship. Binge-watching students may spend less time on academic preparation which results in time displacement [46]. Thus, binge-watching disturbs students’ ability to maintain study-life balance and study plans [32,33]. Additionally, binge-watching may lead to academic procrastination [34] and cognitive distraction [47]. These potential mechanisms should be tested in future studies in children and adolescents. Given that Chinese culture emphasizes academic excellence, Chinese students are often subjected to considerable academic pressure [48]. Low educational efficacy may further exacerbate such academic pressure; in turn, to escape from academic pressure, students may have more binge-watching [48,49]. These relationships should be examined in longitudinal studies. Future work may also compare these relationships with Western cultures where academic excellence is less emphasized.

We found that binge-watching was negatively associated with social support in females but not in males. This finding suggests that binge-watching may have significant social implications for females, as it may reduce their time, opportunities, and effort for both online or offline interpersonal interactions and connections. Female binge-watchers may also be more likely to be perceived as deviant and further isolate them from their significant others. In addition, binge-watching may be a consequence of a lack of social support [50] and females have been consistently found to prioritize interpersonal relationships and help-seeking [51,52]. Qayyoum and Malik [38] also argued that social interaction can be a primary motivation for females to engage in binge-watching. On the other hand, males may tend to fulfil their social needs through online gaming instead of binge-watching. Thus, binge-watching did not significantly affect their social connection and social support. Qualitative studies are needed to further explore this gender difference.

Binge-watching had positive associations with mental health issues in both male and female participants. The results align with some previous studies that investigated college student and adult populations [18,26,53–55]. The study suggests that continuous watching may fail to continuously generate feelings of satisfaction and gratification; instead, binge-watching may act as a stressor and trigger negative emotions [56]. One possible explanation is that video content and TV series on streaming platforms often contain emotionally intense content, such as dramatic or thrilling plots [57], and prolonged exposure to such content can intensify emotional arousal and result in emotional instability and difficulties in emotional regulation [20,58]. In addition, many short-form videos contain unrealistic life content which may lead to watchers’ low life satisfaction and low self-esteem [59]. On the other hand, people with binge watching may have some types of personalities, such as neuroticism, impulsivity, introversion, low self-esteem, and low conscientiousness [4], and maladaptive coping [60]. These traits have been well documented to contribute to mental health problems [58,61,62]. Future work may test how these traits may interact with binge-watching and mental health. The use of qualitative interviews would gain deeper insight into the underlying mechanisms

It is interesting to find that binge-watching was significantly associated with poor sleep quality in males, but not females, after adjusting for age and watching time. This contrasts with the previous findings, which indicated that sleep disturbances were reported primarily by female binge-watchers [38]. Our result may be partly explained by the biological differences in circadian rhythms. A recent study on adolescents found that males have a later melatonin onset and shorter biological night compared to females, making them more vulnerable to sleep disruption from late-night screen use. In contrast, females’ earlier circadian timing and longer biological night may protect them from the same effects, even with similar binge-watching habits [63]. Gender differences in coping and motives may also explain the findings. Male watchers reported higher escapism tendency than females [64]. In other words, males may be more likely to rely on binge-watching to avoid stress, while females might have more effective coping strategies to mitigate the negative effects of binge-watching on sleep quality. In addition, we did not measure watching habits; thus, we did not know whether male participants tended to have a higher frequency of late-night viewing, which can disrupt their sleep patterns more significantly than in females. Males may also tend to watch exciting scenes which can have significant impact on their sleep quality. The potential moderation effects of watching habits and content should be tested to better understand the gender differences in the association between binge-watching and sleep quality.

### Practical implication

The results indicate that an urgent school-based screening and treatment to address binge-watching issues among children and adolescents. Schools should integrate digital literacy education into curriculums to help students develop healthy online habits and understand the risks of binge-watching [65]. Students may enhance their school experience and well-being by promoting sport activities along with face-to-face social engagements, and diverse interest development beyond binge-watching. Parents may provide better supervision to their children and establish shared family rules which should be discussed with their children in order to reduce their binge-watching behaviours. High-quality family time through outdoor activities, family trips or events and exploring natural environments can help build strong parent-child relationships and provide healthy alternatives that prevent children from spending too much time watching screens. At the policy level, the establishment of integrated addiction service centers may be an effective solution to tackle various addictive behaviours within community, including substance use and various behavioural addictions (e.g., binge-watching, gambling, video gaming). These centers can function as professional hubs for sharing evidence-based practices, offering specialized treatment services, and building cross-sector networks to support coordinated prevention campaigns.

While evidence-based intervention for binge-watching is lacking, Cognitive Behavioural Therapy for addictive behaviors, like gaming disorder, has been examined to address impulsivity, anxiety, avoidance, and family and environmental problems in patients, one study designed fourteen 90-minute sessions in a CBT group format, showing reduction in the severity of IGD, impulsivity, and social avoidance [66]. Another growing attention is Collective Motivational Interviewing (CMI) is a novel motivational intervention rooted in the evidence-based strategies and foundational spirit of Motivational Interviewing (MI). It extends the motivational process by involving concerned significant others [67]. While CMI was initially explored in the context of substance use populations with positive outcomes [68]. The approach may also hold promise for adaptation to binge-watching and other forms of problematic digital media use. Group motivational interviewing may also be an alternative for children and adolescents, which has been examined in children, adolescents, and emerging adults with positive outcomes [69]. Future trials should test the efficacy of these interventions on binge-watching. In addition, gender-specific awareness programmes for primary and secondary students are suggested to address potential negative outcomes, as well as the underlying motivations and attitudes associated with binge-watching [70].

### Limitations and future directions

First, our cross-sectional design hindered the development of causal relationships between variables. Second, the convenience sampling approach increased selection bias and limited the generalizability of our findings since it might not account for the variations in school-level differences. Future work may use stratified random sampling to ensure a more representative sample of the population and explore if there are any differences in binge-watching behaviors and their effects among different school types or regions in Hong Kong. Third, instead of using a multi-constructs approach that considers various factors, such as content types, episode consumption, viewing patterns, and related harms, the present study used an inclusive definition of binge-watching by focusing on the duration of viewing time. However, we acknowledge the different types of content (e.g., emotionally charged materials, sports, or comedy), and viewing formats (e.g., long-form episodes, short videos, like reels) may have varying impacts on outcomes. Fourth, some potential confounding factors could affect the research findings including parental supervision, self-regulations, or prior mental health conditions. Future longitudinal studies should include these variables as controls to determine the exact effects observed in the present study. Fifth, multiple outcomes measured in this study (depression, anxiety, stress, sleep quality, loneliness, social support, and educational self-efficacy) increased the chances of Type I errors from multiple comparison tests. The interpretation of results should take into account possible significant inflation. Bonferroni correction may be applied to reduce this risk. In addition, the binary (Yes/No) response to assess whether participants engaged in binge-watching in the past month while neglecting to measure binge-watching frequency or intensity which might not accurately represent its impact on daily responsibilities (e.g., academic, social, or family life). The use of binary predictors may also reduce the statistical power. Furthermore, the study used different time periods for measuring binge-watching behaviour over the past month and mental health symptoms in the past week which might reduce the interpretive value of their association. Future studies should develop and employ timeframe-matched, well-validated, and multi-item scales to validate our results. Qualitative interviews would also provide valuable information on binge-watchers’ experiences to better understand the causes and consequences of binge-watching. The findings of current study stem from Hong Kong Chinese children and adolescents because their high digital consumption is facilitated by strong internet infrastructure, yet academic stress drives parents to stricter control their children’s screen time, in turn, to poor children-parent relationships, which potentially intensifies binge-watching behaviors. Similar socio-economic contexts, strong internet, and academic stress under strict parenting styles may also share comparable findings in regions such as mainland China [71]. The results may not generalize to Western countries like the United States and the United Kingdom, because these countries exhibit distinct cultural norms and parenting practices. A comparative study across cultures could also enhance the external validity of the research findings.

## Conclusion

In conclusion, our findings emphasize the correlation between binge-watching and mental, social, and educational outcomes in children and adolescents with gender-specific impacts. These findings underscore the need for intervention and educational programs focused on enhancing digital use literacy and managing screen time to reduce binge-watching and promote youth well-being.

## Supporting information

S1 Table The Background Information of the Participants. (TIFF)

S2 Table The Association between Binge-watching and Mental, Social and Academic Outcomes. (TIFF)

## References

[1] Schweidel DA, Moe WW. Binge watching and advertising. J Marketing. 2016;80(5):1–19. doi: 10.1509/jm.15.0258

[2] Kuppusamy E, Ganasegaran K, Ganaprakasam C. The prevalence of binge-watching behavior and its association with perceived stress among adolescents during the COVID-19 lockdown: An investigation. MJSSH. 2023;:131–40. doi: 10.33306/mjssh/248

[3] Özkent Y, Açıkel B. The association between binge-watching behavior and psychological problems among adolescents. J Nerv Ment Dis. 2022;210(6):462–7. doi: 10.1097/NMD.0000000000001510 35353791

[4] Starosta JA, Izydorczyk B. Understanding the phenomenon of binge-watching—A systematic review. Int J Environ Res Public Health. 2020;17(12):4469. doi: 10.3390/ijerph17124469 32580289 PMC7344932

[5] Kumar S, Goyal S, Kaur E, Bansal T. Study of relationship between binge watching and motivation for binge watching during COVID-19. J Res Humanities Social Science. 2021;9:5–13.

[6] Anghelcev G, Sar S, Martin JD, Moultrie JL. Binge-watching serial video content: Exploring the subjective phenomenology of the binge-watching experience. Mass Communication and Society. 2020;24(1):130–54. doi: 10.1080/15205436.2020.1811346

[7] Flayelle M, Maurage P, Di Lorenzo KR, Vögele C, Gainsbury SM, Billieux J. Binge-watching: What do we know so far? A first systematic review of the evidence. Curr Addict Rep. 2020;7(1):44–60. doi: 10.1007/s40429-020-00299-8

[8] Wirz DS, Möri M, Ort A, Cordeiro JA, Castro D, Fahr A. The more you watch, the more you get? Re-examining the effects of binge-watching on entertainment experiences. J Media Psychol: Theories, Methods, and Applications. 2023;35(2):99–108. 10.1027/1864-1105/a000355

[9] Sigre-Leirós V, Billieux J, Mohr C, Maurage P, King DL, Schimmenti A, et al. Binge-watching in times of COVID-19: A longitudinal examination of changes in affect and TV series consumption patterns during lockdown. Psychology of Popular Media. 2023;12(2):173–85. doi: 10.1037/ppm0000390

[10] Forte G, Favieri F, Tedeschi D, Casagrande M. Binge-watching: Development and validation of the binge-watching addiction questionnaire. Behav Sci (Basel). 2021;11(2):27. doi: 10.3390/bs11020027 33672253 PMC7926824

[11] Favieri F, Forte G, Tambelli R, Tomai M, Casagrande M. I feel addicted to watching TV series: Association between binge-watching and mental health. PeerJ. 2023;11:e15796. doi: 10.7717/peerj.15796 37576507 PMC10416768

[12] Ramsay D. Confessions of a binge watcher. https://cstonline.net/confessions-of-a-binge-watcher-by-debra-ramsay/ 2013. Accessed 2025 June 9.

[13] Broadband Technology Report. Netflix declares binge watching is the new normal. https://www.broadbandtechreport.com/video/streaming-video/article/16439978/netflix-binge-watching-is-the-new-normal. 2013. Accessed 2025 June 9.

[14] Vaterlaus JM, Spruance LA, Frantz K, Kruger JS. College student television binge watching: Conceptualization, gratifications, and perceived consequences. Social Sci J. 2019;56(4):470–9. doi: 10.1016/j.soscij.2018.10.004

[15] Erickson S, Dal Cin S, Byl H. An experimental examination of binge watching and narrative engagement. Social Sciences. 2019;8(1):19. doi: 10.3390/socsci8010019

[16] Jenner M. Is this TVIV? On Netflix, TVIII and binge-watching. New Media & Society. 2014;18(2):257–73. doi: 10.1177/1461444814541523

[17] Marmet S, Wicki M, Dupuis M, Baggio S, Dufour M, Gatineau C, et al. Associations of binge gaming (5 or more consecutive hours played) with gaming disorder and mental health in young men. J Behav Addict. 2023;12(1):295–301. doi: 10.1556/2006.2022.00086 36592331 PMC10260229

[18] Sun J-J, Chang Y-J. Associations of problematic binge-watching with depression, social interaction anxiety, and loneliness. Int J Environ Res Public Health. 2021;18(3):1168. doi: 10.3390/ijerph18031168 33525732 PMC7908146

[19] Raj N, Ida S. A new era of viewing, binge watching. J Health Sci. 2022;6(S6):7831–40.

[20] Liza LO, Rusandi MA, Situmorang DDB. Binge-watching as one of the new emerging behaviors in the COVID-19 era: Is it dangerous?. J Public Health (Oxf). 2023;45(1):e148–9. doi: 10.1093/pubmed/fdac071 35818289 PMC9384518

[21] Alimoradi Z, Jafari E, Potenza MN, Lin C-Y, Wu C-Y, Pakpour AH. Binge-watching and mental health problems: A systematic review and Meta-analysis. Int J Environmental Res Public Health. 2022;19(15):9707. 10.3390/ijerph19159707 PMC9368441 35955069

[22] Raza SH, Yousaf M, Sohail F, Munawar R, Ogadimma EC, Siang JMLD. Investigating binge-watching adverse mental health outcomes during COVID-19 pandemic: Moderating role of screen time for web series using online streaming. Psychol Res Behav Manag. 2021;14:1615–29. doi: 10.2147/PRBM.S328416 34675702 PMC8518137

[23] Utaibah Naseer SS Kiran Jafferani. Rise of Netflix during COVID-19 pandemic & its impact on youth. PJIA. 2022;5(4). doi: 10.52337/pjia.v5i4.696

[24] Chang Y-J, Peng C-Y. Exploring experiences of binge-watching and perceived addictiveness among binge-watchers: A qualitative study. BMC Public Health. 2022;22(1):2285. doi: 10.1186/s12889-022-14789-z 36474226 PMC9724272

[25] Anghelcev G, Sar S, Martin J, Moultrie JL. Is heavy binge-watching a socially driven behaviour? Exploring differences between heavy, regular and non-binge-watchers. J Digital Media & Policy. 2022;13(2):201–21. doi: 10.1386/jdmp_00035_1

[26] Singh DrR. Binge watching on OTT platforms: Loneliness in adolescents. IJSSHR. 2022;05(08):3720–5. doi: 10.47191/ijsshr/v5-i8-50

[27] Wong RS, Tung KTS, Rao N, Leung C, Hui ANN, Tso WWY, et al. Parent technology use, parent-child interaction, child screen time, and child psychosocial problems among disadvantaged families. J Pediatr. 2020;226:258–65. doi: 10.1016/j.jpeds.2020.07.006 32629010

[28] Beyens I, Beullens K. Parent–child conflict about children’s tablet use: The role of parental mediation. New Media & Society. 2016;19(12):2075–93. doi: 10.1177/1461444816655099

[29] Sanz-Martín D, Melguizo-Ibáñez E, Ruiz-Tendero G, Ubago-Jiménez JL. An explanatory model of the relationships between physical activity, social support and screen time among adolescents. Int J Environ Res Public Health. 2022;19(12):7463. doi: 10.3390/ijerph19127463 35742710 PMC9223611

[30] Singh M. Screen time usage and its relations with social support, perceived distress and psychological well being among indian youth. IJRAR-International Journal of Research and Analytical Reviews. 2022;9(4):634–70.

[31] Zimmerman BJ. Self-efficacy: An essential motive to learn. In: Bandura A, editor. Self-efficacy in changing societies. New York: Cambridge University Press. 1995. p. 202–31.

[32] Faminiano MT, Yango AR. Netflix viewing habit, grammatical competence, and academic self-efficacy of selected senior high school students in Tagaytay City, Cavite. TSSJ. 2023;44:316–31. doi: 10.47577/tssj.v44i1.8947

[33] Steffanie Evelyn Yolie, Rahmah Hastuti. The relationship between binge-watching habits and academic procrastination among college students. JCSI. 2024;5(2):97–105. doi: 10.58915/jcsi.v5i2.1096

[34] Paulus AM, Aziz A. Binge watching, compensatory health beliefs and academic procrastination among university students. J Behavioural Sciences. 2023;33(1):25–47.

[35] Chambliss C, Gartenberg C, Honrychs D, Elko M, Match R, McGill S. Distracted by binge-watching: Sources of academic and social disruption in students. ARC J Pediatrics. 2017;3(1):14–7.

[36] Prakasha GS, Rangasamy S, Jahnavitha V, Dlima S. Students’ usage of Over-the-top (OTT) streaming platforms affecting their academic and socio-demographic profile. IJTEI. 2024;10(1):124–43. doi: 10.24310/ijtei.101.2024.17082

[37] Moore AE. Binge watching: Exploring the relationship of binge watched television genres and colleges at Clemson University. Graduate Research and Discovery Symposium (GRADS). 2015. 138.

[38] Qayyoum H, Malik Q-A. Gender differences in binge-watching by teenagers: A uses and gratification analysis. JSSH. 2023;31(1):435–50. doi: 10.47836/pjssh.31.1.23

[39] Viola JK. Educational self-efficacy scale. https://www.imperial.ac.uk/education-research/evaluation/what-can-i-evaluate/self-efficacy/tools-for-assessing-self-efficacy/educational-self-efficacy-scale/ 2021. Accessed 2025 June 1.

[40] Zimet GD, Dahlem NW, Zimet SG, Farley GK. The multidimensional scale of perceived social support. J Personality Assessment. 1988;52(1):30–41. doi: 10.1207/s15327752jpa5201_2 2280326

[41] Chou K-L. Assessing Chinese adolescents’ social support: The multidimensional scale of perceived social support. Personality and Individual Differences. 2000;28(2):299–307. doi: 10.1016/s0191-8869(99)00098-7

[42] Hughes ME, Waite LJ, Hawkley LC, Cacioppo JT. A short scale for measuring loneliness in large surveys: Results from two population-based studies. Res Aging. 2004;26(6):655–72. doi: 10.1177/0164027504268574 18504506 PMC2394670

[43] Lovibond PF, Lovibond SH. The structure of negative emotional states: Comparison of the Depression Anxiety Stress Scales (DASS) with the Beck Depression and Anxiety Inventories. Behav Res Ther. 1995;33(3):335–43. doi: 10.1016/0005-7967(94)00075-u 7726811

[44] Zanon C, Brenner RE, Baptista MN, Vogel DL, Rubin M, Al-Darmaki FR, et al. Examining the dimensionality, reliability, and invariance of the Depression, Anxiety, and Stress Scale-21 (DASS-21) across eight countries. Assessment. 2021;28(6):1531–44. doi: 10.1177/1073191119887449 31916468

[45] Farrahi Moghaddam J, Nakhaee N, Sheibani V, Garrusi B, Amirkafi A. Reliability and validity of the Persian version of the Pittsburgh Sleep Quality Index (PSQI-P). Sleep Breath. 2012;16(1):79–82. doi: 10.1007/s11325-010-0478-5 21614577

[46] Osman D, Farag SM, Mansur GM. The effects of Netflix binge watching on Egyptian teenagers’ academic achievement. Egyptian J Public Opinion Res. 2022;21(2):161–89.

[47] Jacobsen WC, Forste R. The wired generation: academic and social outcomes of electronic media use among university students. Cyberpsychol Behav Soc Netw. 2011;14(5):275–80. doi: 10.1089/cyber.2010.0135 20961220

[48] Shek DTL, Chai W. The impact of positive youth development attributes and life satisfaction on academic well-being: A longitudinal mediation study. Front Psychol. 2020;11:2126. doi: 10.3389/fpsyg.2020.02126 32982869 PMC7490328

[49] Zhang K, Song Y. Social media use in Hong Kong. In: Huang Y, Song Y, editors. Evolving landscape of media and communication in Hong Kong. Hong Kong: City University of Hong Kong Press. 2018.

[50] Starosta J, Izydorczyk B, Lizińczyk S. Characteristics of people’s binge-watching behavior in the “entering into early adulthood” period of life. Health Psychology Report. 2019;7(2):149–64. doi: 10.5114/hpr.2019.83025

[51] Liddon L, Kingerlee R, Barry JA. Gender differences in preferences for psychological treatment, coping strategies, and triggers to help-seeking. Br J Clin Psychol. 2018;57(1):42–58. doi: 10.1111/bjc.12147 28691375

[52] Rudolph KD. Gender differences in emotional responses to interpersonal stress during adolescence. J Adolesc Health. 2002;30(4 Suppl):3–13. doi: 10.1016/s1054-139x(01)00383-4 11943569

[53] Tolba AA, Zoghaib SZ. Understanding the binge-watching phenomenon on Netflix and its association with depression and loneliness in Egyptian adults. Media Watch. 2022;13(3):264–79. doi: 10.1177/09760911221117339

[54] Gabbiadini A, Baldissarri C, Valtorta RR, Durante F, Mari S. Loneliness, escapism, and identification with media characters: An exploration of the psychological factors underlying binge-watching tendency. Front Psychol. 2021;12:785970. doi: 10.3389/fpsyg.2021.785970 35069369 PMC8771202

[55] Yu H, Alizadeh F. Online binge-watching among Chinese college students: Implications for loneliness, anxiety, and depression. Psychol Res Behav Manag. 2024;17:295–303. doi: 10.2147/PRBM.S447311 38292254 PMC10826546

[56] Aghababian AH, Sadler JR, Jansen E, Thapaliya G, Smith KR, Carnell S. Binge watching during COVID-19: Associations with stress and body weight. Nutrients. 2021;13(10):3418. doi: 10.3390/nu13103418 34684420 PMC8539795

[57] Granow VC, Reinecke L, Ziegele M. Binge-watching and psychological well-being: Media use between lack of control and perceived autonomy. Communication Research Reports. 2018;35(5):392–401. doi: 10.1080/08824096.2018.1525347

[58] Starosta J, Izydorczyk B, Dobrowolska M. Personality traits and motivation as factors associated with symptoms of problematic binge-watching. Sustainability. 2020;12(14):5810. doi: 10.3390/su12145810

[59] Asgher S, Gohar I. Binge watching on internet television networks & its effects on youth. J Media Communication. 2022;3(1):52–64.

[60] Verrastro V, Calaresi D, Giordano F, Saladino V. Vulnerable narcissism and emotion dysregulation as mediators in the link between childhood emotional abuse and binge watching. Eur J Investig Health Psychol Educ. 2024;14(10):2628–41. doi: 10.3390/ejihpe14100173 39452168 PMC11507281

[61] Forte G, Favieri F, Casagrande M, Tambelli R. Personality and behavioral inhibition/activation systems in behavioral addiction: Analysis of binge-watching. Int J Environ Res Public Health. 2023;20(2):1622. doi: 10.3390/ijerph20021622 36674381 PMC9863166

[62] Kang W, Steffens F, Pineda S, Widuch K, Malvaso A. Personality traits and dimensions of mental health. Sci Rep. 2023;13(1):7091. doi: 10.1038/s41598-023-33996-1 37127723 PMC10151354

[63] Duston A, Holtman S, Bowen AE, Cree MG, Nadeau K, Wright KP Jr, et al. Sex differences in circadian timing and biological night in adolescents. J Biol Rhythms. 2025;40(1):7–18. doi: 10.1177/07487304241309165 39876068 PMC12285599

[64] Alfonsi V, Varallo G, Scarpelli S, Gorgoni M, Filosa M, De Gennaro L, et al. “This is the last episode”: The association between problematic binge-watching and loneliness, emotion regulation, and sleep-related factors in poor sleepers. J Sleep Res. 2023;32(1):e13747. doi: 10.1111/jsr.13747 36254098 PMC10078456

[65] Taiwade H, Yerwarkar A, Sewatkar G, Mandape M, Patle M, Koli S. Decreasing the screen time on social media using time limitations. Int J Ad Res Sci Communic Technol. 2022;2(5):229-33. doi: 10.48175/ijarsct-4802

[66] Han J, Seo Y, Hwang H, Kim SM, Han DH. Efficacy of cognitive behavioural therapy for internet gaming disorder. Clin Psychol Psychother. 2020;27(2):203–13. doi: 10.1002/cpp.2419 31881100

[67] Tse N, Tse S, Wong P, Adams P. Collective motivational interviewing for substance use problems: Concept and implications. Int J Ment Health Addiction. 2022;21(4):2538–55. doi: 10.1007/s11469-021-00736-3

[68] Tse N, Tse S, Wong PWC. Collective motivational interviewing for individuals with drug use problems: A pre-post-follow-up, uncontrolled pilot study. Int J Environ Res Public Health. 2022;19(23):16344. doi: 10.3390/ijerph192316344 36498414 PMC9737559

[69] Tse N, Siu A, Tsang S, Jensen MP. Group motivational interviewing for adolescents at risk of internet gaming disorder: A mixed-methods preliminary evaluation. Clin Soc Work J. 2024. doi: 10.1007/s10615-024-00968-5

[70] Aytas M, Topatan IB. Determining factors of university students’ binge-watching attitudes. Heliyon. 2024;10(20):e39642. doi: 10.1016/j.heliyon.2024.e39642 39498067 PMC11532883

[71] Liu S, Lan Y, Chen B, He G, Jia Y. Smartphone use time and total screen time among students aged 10-19 and the effects on academic stress: A large longitudinal cohort study in Shanghai, China. Front Public Health. 2022;10:869218. doi: 10.3389/fpubh.2022.869218 35655462 PMC9152090

