# Supplementary figures and images for: Binge-watching and gender-specific effects on academic, social, and mental well-being in children and adolescents
Source: PLoS One. 2025 Aug 22;20(8):e0329655. doi: 10.1371/journal.pone.0329655 (PMC12373196; doi:10.1371/journal.pone.0329655)

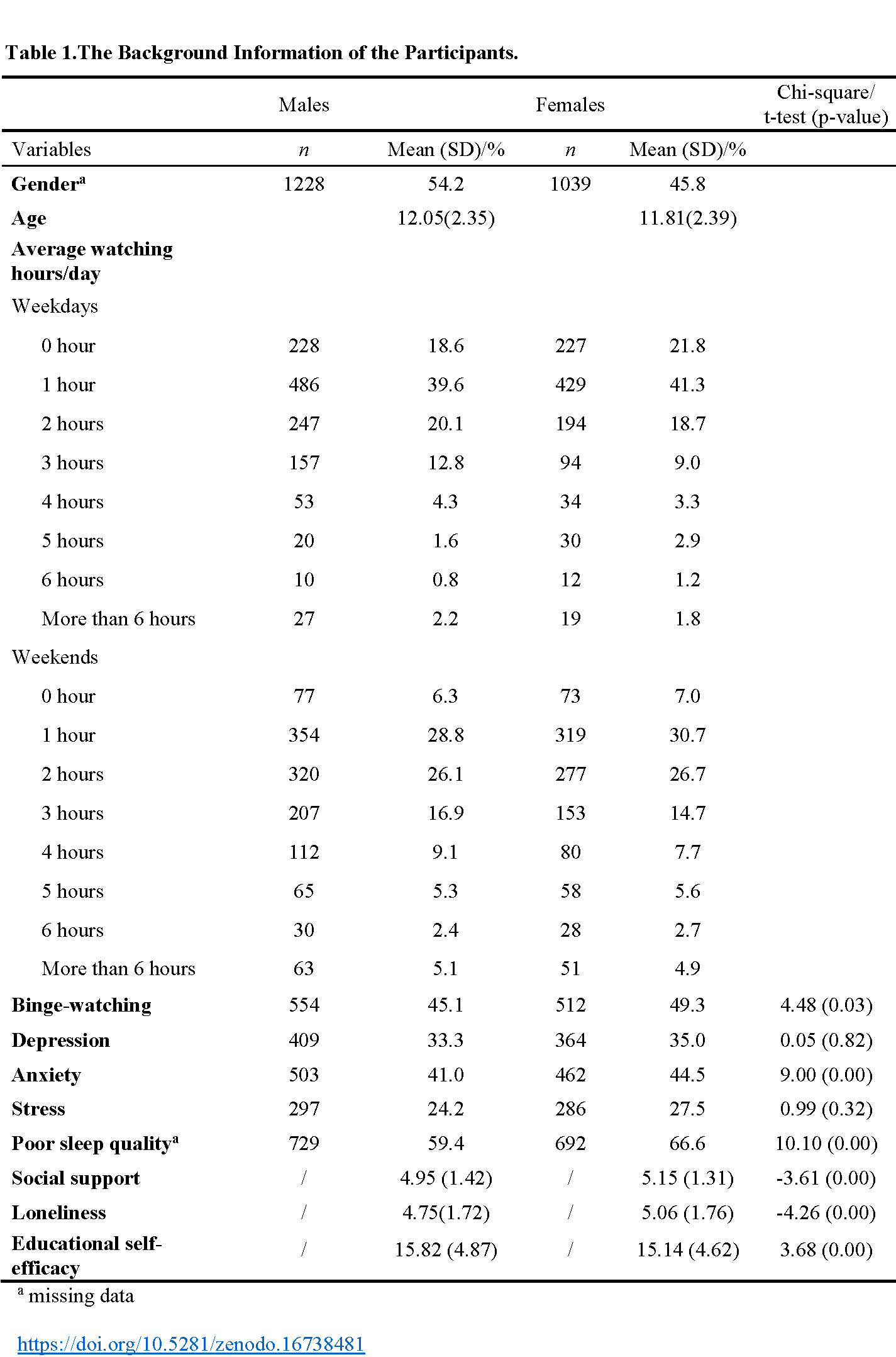

Supplement: S1 Table — (TIFF) [file pone.0329655.s001.tiff]

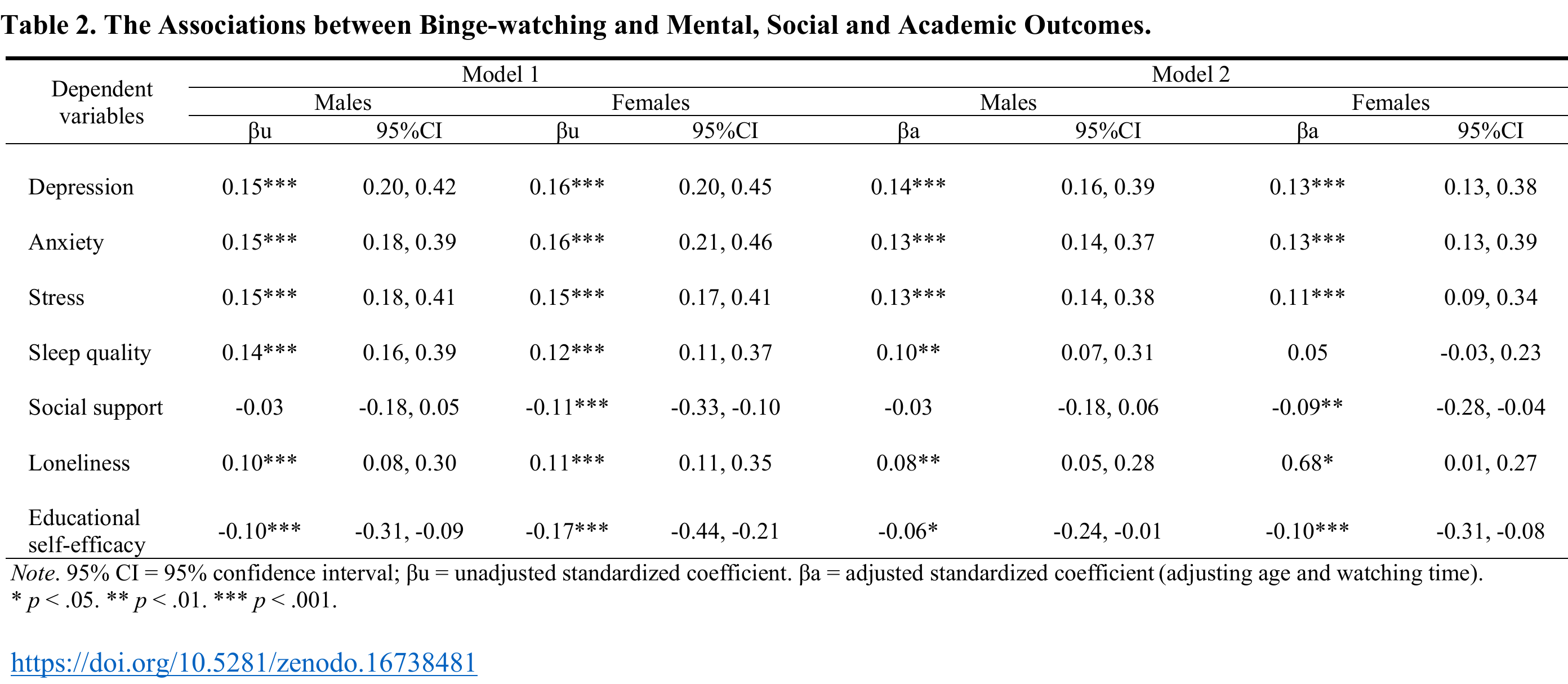

Supplement: S2 Table — (TIFF) [file pone.0329655.s002.tiff]
